# Prematurity and low birth weight: geospatial analysis and recent trends

**DOI:** 10.1186/s40748-022-00137-x

**Published:** 2022-04-29

**Authors:** Nicholas Peterman, Bradley Kaptur, Morgan Lewis, Lindsey Ades, Kristine Carpenter

**Affiliations:** 1 Carle Illinois College of Medicine, University of Illinois Urbana-Champaign, IL Champaign, USA; 2 Carle Foundation Hospital, IL Urbana, USA

**Keywords:** Prematurity, Low birth weight, Geospatial, Cluster, Neonatal

## Abstract

Prematurity and low birth weight are of concern in neonatal health. In this work, geospatial analysis was performed to identify the existence of statistically significant clusters of prematurity and low birth weight using Moran’s I. Data was obtained from March of Dimes and the National Center for Health Statistics for the years 2015 to 2019. Analysis demonstrated the presence of hotspot (High-High) and coldspot (Low-Low) geographic clusters of these variables in regions across the United States. Additionally, factorial ANOVA was performed, and revealed the significance of demographic variables of interest. Given the strong relationship between these two variables, regions that are hotspots for one variable, but not the other, are of particular interest for further study.

Letter to the editor

Dear Editor,

It has been previously established that prematurity (PM) and low birth weight (LBW) are of concern when assessing neonatal health: Prior works have demonstrated the role of these variables in predicting neonatal morbidity and mortality [1]. Additionally, previous research has shown the role of both the health of the mother and her socioeconomic environment in the prevalence of these two conditions [2]. We aimed to use geospatial analysis techniques to identify whether statistically significant clusters of PM (< 37 weeks) and LBW (< 5.5 lbs) exist on a nationwide level and to further explore the socioeconomic determinants associated with those clusters.

We used birth and C-section data from the March of Dimes and the National Center for Health Statistics during the years 2015–2019 across 3105 US counties [3]. Moran’s I statistic was calculated to categorize individual counties as either Not Significant or as one of 4 statistically significant (*p* < 0.05) cluster classifications: High-High (H-H), High-Low (H-L), Low-High (L-H), Low-Low (L-L) [4]. In this attribution system, the first term designates the relative value of a given county compared to the national average; the second attribute reflects the relative value of neighboring counties compared to the national average. Demographic data was obtained from the American Community Survey (US Census Bureau). Factorial ANOVA was performed to evaluate the significance of contributory socioeconomic variables of interest at a significance level of 0.001.

Visualization of the cluster designations at the county level demonstrated clear geographic trends (Fig. 1). For both the PM and LBW analyses, there was an expansive H-H cluster that was persistent across the Southern states. There were 3 distinct expansive L-L clusters encompassing the New England states, the Midwest, and the Pacific Northwest. A LBW H-H cluster encompassed Colorado and northern New Mexico, yet this was not seen in the PM analysis. Similarly, multiple significant PM H-H clusters were identified in Texas, but not LBW clusters. Factorial ANOVA across clusters revealed significant contributions of various socioeconomic factors at a significance level of 0.001 for both the PM and LBW analyses (Tables 1 and 2).

**Fig. 1:**
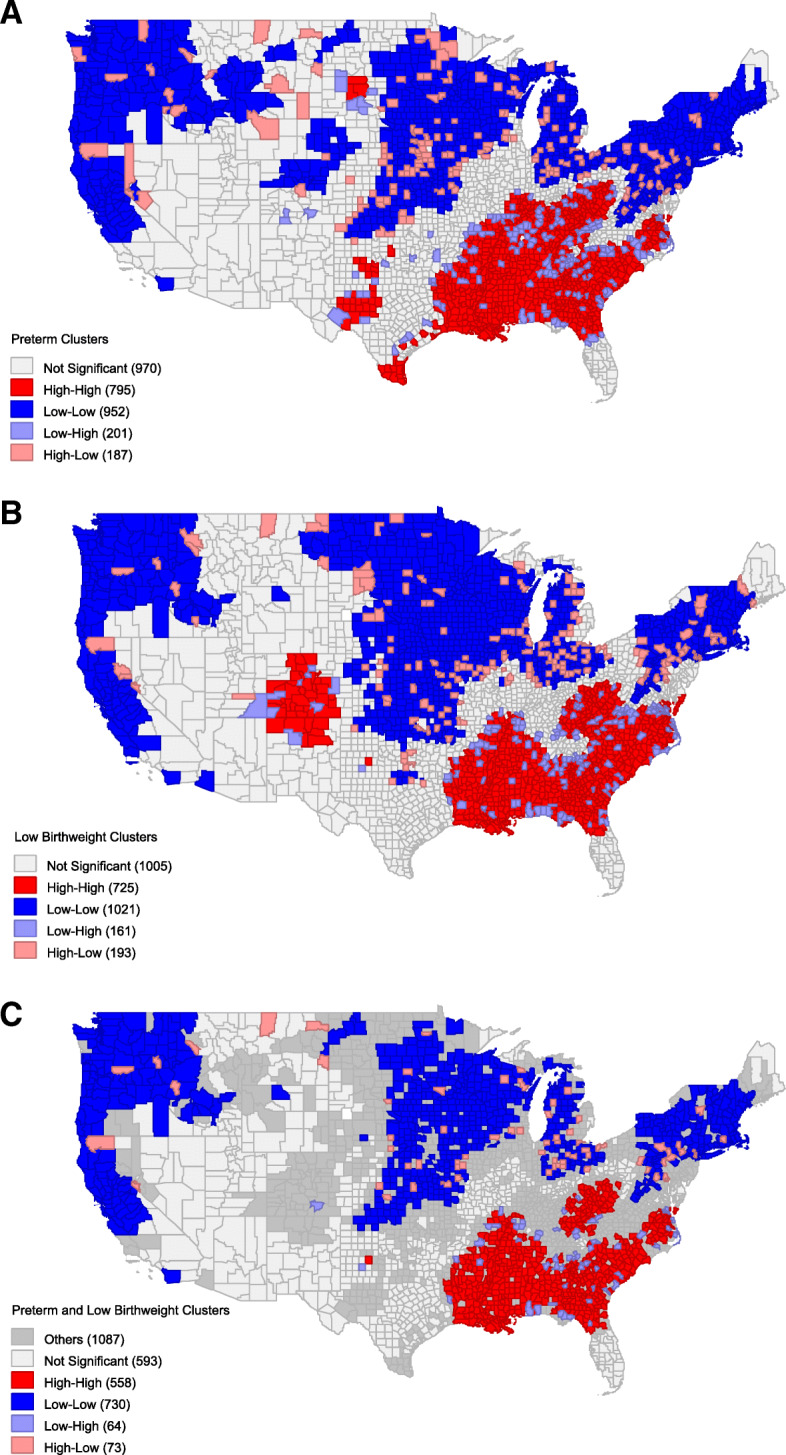
Geospatial mappings and analysis for (**A**) prematurity, (**B**) low birth weight, and (**C**) joint prematurity and low birth weight. Color designations reflect Moran’s I spatial categorizations. For joint mappings, High and Low designations represent agreement and Other represents areas of disagreement between the two variables

**Table 1:** Factorial ANOVA across preterm birth clusters. Asterisks reflect significance at a significance level of 0.001

ANOVA: Cluster Analysis of Preterm Birth									
**Cluster**	**High-High**		**Low-Low**		**Low-High**		**High-Low**		***P-value***
**Counties per Cluster**	**795**		**952**		**201**		**187**		
**Demographic Variable**	Mean	SD	Mean	SD	Mean	SD	Mean	SD	
Population	64496.61	196449.9	126214.1	280923.7	69071.4	140112.1	106369.1	252554.9	6.09E-07*
% White	72	20.86	89.12	10.51	83.49	13.68	86.84	15.03	9.04E-103*
% Black	22.68	20.48	3.12	5.34	10.52	12.53	4.47	7.67	1.40E-166*
% American Indian	0.83	5	1.12	3.04	0.62	1.26	3.35	12.27	1.97E-08*
% Asian	0.78	1.04	1.99	3.58	1.07	1.61	1.18	1.58	2.72E-21*
% Hispanic	6.51	12.41	7.83	9.97	8.43	12.45	7.56	8.57	0.04073
Median Household Income	44685.49	10668.47	60557.75	14883.34	50749.5	13795.23	54713.7	10114.64	3.72E-124*
% With SNAP Benefits in Past Year	17.55	6.56	9.85	4.5	13.62	5.53	11.49	5.22	2.63E-157*
% With Health Insurance	88.98	4.49	93.01	3.5	89.25	4.19	91.71	5.6	1.83E-87*
% With Public Health Insurance	43.65	8.58	37.17	8.36	40.37	9.13	38.73	7.59	6.85E-53*
% Families in Poverty	15.21	5.77	7.66	3.07	11.77	4.67	9.32	4.37	1.98E-212*
% Households: Married	47.41	7.25	51.96	5.66	51.98	6.02	49.85	6.39	6.67E-49*
% Households: Single Parent	6.25	2.48	3.81	1.38	4.78	1.91	4.47	1.74	1.74E-130*
% Births: Unmarried	44.31	20.3	29.41	14.9	34.19	19.67	37.7	16.94	1.82E-62*
% 25 + Year Old: Bachelor’s Degree or Beyond	17.28	7.12	26.09	10.6	21.12	10.63	21.71	6.45	2.55E-80*
% Households: Spanish Speaking	4.95	9.82	5.43	8.07	6.13	9.05	4.98	6.72	0.320591
Population Density	148.58	363.72	486.89	3125.64	172.81	333.32	357.03	1187.19	0.007615
2013 Rural Urban Cont. Code	4.99	2.57	4.79	2.75	4.82	2.75	5.17	2.7	0.215512

**Table 2:** Factorial ANOVA across low birth weight clusters. Asterisks reflect significance at a significance level of 0.001

ANOVA: Cluster Analysis of Low Birth Weight									
**Cluster**	**High-High**		**Low-Low**		**Low-High**		**High-Low**		***P-value***
**Counties per Cluster**	**725**		**1021**		**161**		**193**		
**Demographic Variable**	Mean	SD	Mean	SD	Mean	SD	Mean	SD	
Population	66370.72	123966.7	110074.2	399492.9	69749.57	115894.8	195134.5	519333.4	1.07E-05*
% White	69.22	20.42	89.72	10.22	84.58	11.14	84.67	17.68	2.92E-143*
% Black	25.77	20.4	2.11	2.85	9.72	9.61	5.48	7.74	1.15E-248*
% American Indian	0.62	2.36	1.94	6.45	0.94	4.17	3.72	14.46	8.65E-09*
% Asian	0.87	1.14	1.73	3.18	1.22	1.76	1.51	2.72	6.69E-11*
% Hispanic	5.68	8.33	7.31	9.54	6.07	7.25	7.44	9.2	0.000999*
Median Household Income	44430.52	11380.09	59225.79	13116.82	53804.39	16338.27	53001.26	9956.9	4.56E-112*
% With SNAP Benefits in Past Year	17.75	6.58	9.72	4.38	12.9	5.84	12.82	5.91	2.66E-163*
% With Health Insurance	89.15	3.51	92.75	4.13	90.27	3.63	91.04	6.11	1.73E-66*
% With Public Health Insurance	44.25	8.61	36.8	7.97	39.34	9.74	39.87	7	5.00E-69*
% Families in Poverty	15.34	5.72	7.82	3.35	10.77	5.01	10.51	5.57	8.77E-194*
% Households: Married	46.35	7.22	52.31	5.17	52.51	6.09	48.6	6.51	6.01E-86*
% Households: Single Parent	6.3	2.51	3.81	1.35	4.66	1.7	4.76	2.2	3.31E-132*
% Births: Unmarried	45.05	20.83	29.92	14.97	31.85	18.82	36.18	16.1	9.78E-65*
% 25 + Year Old: Bachelor’s Degree or Beyond	18.86	9.19	24.62	9.28	22.76	11.82	21.86	7.72	1.34E-33*
% Households: Spanish Speaking	4.11	5.57	5.02	7.7	4.08	4.45	5.21	7.72	0.0179
Population Density	177.29	446.45	287.84	2608.33	194.83	331.32	776.73	3267.5	0.00491
2013 Rural Urban Cont. Code	4.89	2.54	5.11	2.74	4.3	2.86	4.97	2.81	0.004411

PM and LBW have previously been demonstrated to have a strong relationship, so it is unsurprising that the identified spatial clusters of these variables have substantial overlap, However, what is of particular interest are the regions that are clusters for one variable but not the other. For instance, there are regions of Texas where several counties are significantly higher in PM but not LBW. Conversely, there is a large region of Colorado where there is a substantial incidence of LBW despite that region not having high prematurity. Given that factors traditionally associated with prematurity would not explain this increase, it is important to look for other explanations. The Colorado Department of Public Health has previously proposed the contribution of high altitude to pregnancy-induced hypertension as a possible explanatory factor [5]. The inverse relationship in Texas is harder to attribute to an isolated cause; though, the prevalence of large, medically underserved immigrant communities in the identified regions is likely a contributing factor. The ANOVA findings in this study underscore the importance of many socioeconomic factors that differentiate the clusters, including race and various economic markers (e.g., SNAP, insurance type, educational status). Interestingly, the rural/urban character between clusters did not significantly differ in this analysis.

Since PM and LBW demonstrate similar geospatial patterns across the United States, and a strong relationship exists between these two factors, regions that are high in one variable and not the other are of particular interest for further study.

## Data Availability

This study uses publicly available data that is available through March of Dimes and the National Center for Health Statistics at https://www.marchofdimes.org/peristats/Peristats.aspx. Demographic data was obtained through the US Census Bureau American Community Survey (ACS), which is available at https://www.census.gov/programs-surveys/acs/data.html. Definitions of race and ethnicity used in this work are derived from those used in the ACS.

## Abbreviations

LBW: Low birth weight
PM: Prematurity
H-H: High-High
H-L: High-Low
L-L: Low-Low
L-H: Low-High
ANOVA: Analysis of Variance
